# Comparison of a novel algorithm quantitatively estimating epifascial fibrosis in three-dimensional computed tomography images to other clinical lymphedema grading methods

**DOI:** 10.1371/journal.pone.0224457

**Published:** 2019-12-10

**Authors:** Kyo-in Koo, Myoung-Hwan Ko, Yongkwan Lee, Hye Won Son, Suwon Lee, Chang Ho Hwang

**Affiliations:** 1 Department of Biomedical Engineering, School of Electrical Engineering, University of Ulsan, Ulsan, Republic of Korea; 2 Department of Physical Medicine and Rehabilitation, Jeonbuk National University Medical School and Research Institute of Clinical Medicine of Jeonbuk National University-Biomedical Research Institute of Jeonbuk National University Hospital, Jeonbuk, Republic of Korea; 3 Department of Physical Medicine and Rehabilitation, Ulsan University Hospital, University of Ulsan College of Medicine, Ulsan, Republic of Korea; University Magna Graecia of Catanzaro, ITALY

## Abstract

No method has yet been approved for detecting lymphedema fibrosis before its progression. This study assessed the feasibility of computed tomography-based estimation of fibrosis. This observational, cross-sectional study included patients with lymphedema affecting one limb. Three types (maximum, mean, minimum) of computed tomography reticulation indexes were digitally calculated from trans-axial images using absorptive values, and the computed tomography reticulation indexes compared with clinical scales and measurements. Of 326 patients evaluated by at least one of lymphoscintigraphy, bio-electrical impedance, and computed tomography, 24 were evaluated by all three. The mean number of computed tomography scans in these patients was 109. Sixteen patients had breast cancer, seven had gynecologic cancers, and one had primary lymphedema. Mean computed tomography reticulation index (*r* = 0.52, *p* < 0.01) and maximal computed tomography reticulation index (*r* = 0.45, *p* < 0.05) were significantly associated with time from initial limb swelling to computed tomography. Mean computed tomography reticulation index (*r* = 0.86, *p* < 0.01), minimal computed tomography reticulation index (*r* = 0.79, *p* < 0.01), and maximal computed tomography reticulation index (*r* = 0.68, *p* < 0.01) were significantly associated with International Society of Lymphedema substage. Minimal computed tomography reticulation index correlated with 1-kHz-based bio-electrical impedance ratio (*r* = -0.46, *p* < 0.05) and with standardized proximal limb circumference difference ratio (*r* = 0.45, *p* < 0.05) of both limbs. Maximal computed tomography reticulation index had a sensitivity of 0.78, specificity of 0.60, and areas under the curve of 0.66 in detecting lymphoscintigraphic stage IV. The algorithm utilizing three-dimensional computed tomography images of epifascial fibrosis may be used as a marker for lymphedema duration, limb swelling, International Society of Lymphedema substage, and interstitial lymphatic fluids of lymphedema. The current approach shows promise in providing an additional method to assist in characterizing and monitoring lymphedema patients.

## Introduction

Lymphedema can occur in various oncologic situations, including in patients with lymph node metastases and those undergoing radio-therapy, as well as following surgery for gynecologic, breast, prostate, and head and neck cancers.[1–7] However, the lack of standard measurements has been a barrier to early detection and successful management,[5] and even three-dimensional perometry findings do not correlate with significant deterioration in patient quality of life (QOL).[8] Because swelling is progressive and can differ in degree between the subfascial muscular compartment and epifascial skin and subcutaneous compartments,[9] internal changes, as well as external swelling, should be determined.

The accumulation of interstitial lymphatic fluid can result in the increased release of pro- inflammatory cytokines, including transforming growth factor (TGF)-β1, tumor necrosis factor (TNF)-α, interleukin (IL)-6, and IL-8. This results in the deposition of collagen and extracellular matrix, leading to macroscopic fibrosis.[10, 11] Honeycomb-patterned trabecular structures in the subcutaneous compartment have been observed in patients with lymphedema,[12] not in those with venous edema or lipodystrophy, with these epifascial reticulations be markers of lymphedema-induced fibrosis.[13, 14] Meanwhile, the appearance of these structures does not mean a complete lack of response to rehabilitation management,[15, 16] suggesting that it can still bear the potential benefit for early detection of lymphedema-induced fibrosis. So, the lack of standard measurements inhibiting early monitoring and successful management should be solved.[5]

Narrowing the width of absorptive values (Hounsfield unit [HU]) in computed tomography (CT) can enhance the sensitivity of this method in differentiating among organs.[17] The absorptive values of the connective tissues may depend on their relative composition, such that digital subtraction from CT images may enable the visualization of lymphedema-induced fibrosis. This study therefore compared several values obtained from CT images with clinical scales and contributing factors.

## Materials and methods

This observational, cross-sectional, cohort trial involved patients evaluated from January 2017 to March 2018 at a tertiary medical center/teaching university hospital. The study protocol was approved by the Ulsan University Hospital Institutional Review Board and was registered at the Protocol Registration and Results System (PRS), www.clinicaltrials.gov, (NCT03523494: https://register.clinicaltrials.gov/prs/app/action/SelectProtocol?sid=S0007XEL&selectaction=Edit&uid=U00014UZ&ts=5&cx=-g6se3t). The study was conducted according to the Declaration of Helsinki. It included patients who were clinically diagnosed with lymphedema involving a unilateral limb and who underwent CT, multi-frequency bio-electric impedance (BEI) analysis, and lymphoscintigraphy (LSG). The individual in this manuscript has given written informed consent (as outlined in PLOS consent form) to publish these case details. Data obtained closest to the CT scanning date as possible were evaluated. Patients with bilateral involvement, deep vein thrombosis, local infection or vascular diseases were excluded.

### Image processing of CT

**Fig 1:**
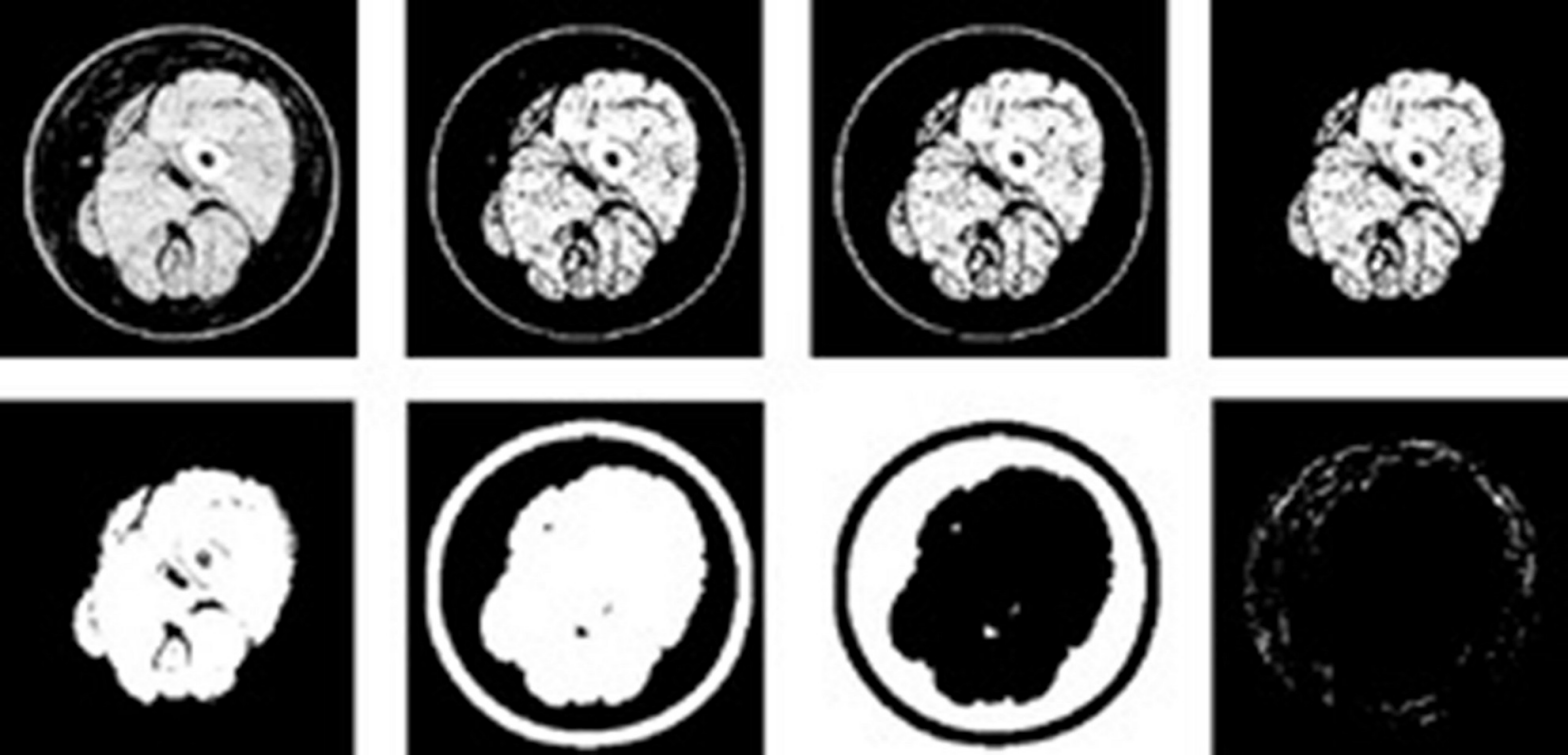
The images under the proposed image processing procedures. The original CT image of the affected limb (the first image; the farthest left one in the upper row), six intermediate images and the final image (farthest right in the lower row) are shown. The algorithm eliminated pixels from -50 Hounsfield unit (HU) to 26 HU (image 2), and removed any remained noise using a simple opening filter and manual reoperation (image 3). The outmost skin was removed based on its location (image 4). The remained pattern was processed as one connected region using the closing filter (image 5). Outside of this one connected region, the eliminated skin line was added as one outside thick line (image 6). This merged morphological pattern was converted by the black-white reversion operation. (image 7). The inversed pattern subtracted from the original CT image yielding a donut-shaped region between the skin and the muscle. (image 8). The nonblack pixels of the final image were regarded as epifascial lymph-proliferative fibrosis.

Patients were requested to maintain an anatomically neutral position, fully extending both arms if necessary. CT simultaneously scanned both limbs from the point of leg separation to 5 cm above the lateral malleolus or from the distal ends of both clavicles to the wrist crease.[9] The proposed image processing algorithm proceeded following sequences with the scanned CT images to recognize the reticulated pixels, as summarized in Fig 1. In order to select only the skin, muscle, and bone, pixels from -50 HU to 26 HU were eliminated [17]. Any remained noise was removed by a simple opening filter, and then removed again by manual operation. The outmost skin was removed based on its location. The remained pattern was processed as one connected region by the closing filter. Outside of this one connected region, the eliminated skin line added as one outside thick line. This merged morphological pattern was converted by the black-white reversion operation. The inversed pattern subtracted from the original CT image yielding a donut-shaped region between the skin and the muscle. The number of remained nonblack pixels in the donut-shaped region was counted as epifascial reticulations. The CT reticulation index (CTRI) was defined as the summated ratio of the number of pixels of the affected limb to the number of pixels of the unaffected limb over the whole length of the scanned limbs. Meanwhile, each patient was scanned with multiple cross-sectional scans and the first step of the imaging processing included manual reoperation, following noise removal by a simple opening filter. As the result, each value of CTRI might be varied whenever image processing was performed. To minimize effect on the results by this variation, authors conducted 10 times of the image processing and calculated three types of CTRIs (maximum, mean, and minimum).

### International Society of Lymphology (ISL) classification

The ISL subscale, composed of six grades, has been described previously.[18]

### 1 kHz-based BEI

Patients were instructed to maintain their regular diets the day before the evaluation and to rest in a supine position with no movement for 20 minutes before the evaluation. Results were recorded using the Direct Segmental Measurement BEI Analysis Method. The value of the affected limb was subtracted from the value of the unaffected limb, with the result divided by the value of the unaffected limb.

### LSG stage

Patients in the supine position underwent two-compartment LSG 1 and 2 hours after Techne Phytate injection into the second and third webs. The function of the epifascial and subfascial lymphatic systems was staged as described,[19–21] with stage I (no lymph stasis) indicating normal epifascial and subfascial systems; stage II (slow lymphatic transport) indicating visible but dysfunctional epifascial lymph nodes; stage III (lymphatic blockage) indicating dermal back flow and no visible epifascial lymph nodes, but visible subfascial lymph nodes; and stage IV (no transport) indicating visible dermal back flow and no visible epifascial or subfascial lymph nodes.

### Standardized circumferences difference ratio (SCDR)

The circumference of both limbs 5 cm above and below the mid-antecubital or mid-popliteal fossa was measured using a nonelastic 1-mm polyvinyl chloride fiberglass tape, with the patient in the supine position. The difference between the two limbs was divided by the circumference of the unaffected limb.

### Statistical analysis

All statistical analyses were performed using the Statistical Package for the Social Sciences, version 24 (SPSS Inc., Chicago, USA). The Kolmogorov-Smirnov or Shapiro-Wilk test was used to assess kurtosis, skewness, and normal distribution. CTRIs were analyzed by Spearman correlation analysis, after log transformation in the case of non-normal distributions, or by the Mann-Whitney U test. The diagnostic ability of the CTRIs was evaluated by receiver operating characteristics (ROC) curve analysis.

## Results

### Demographic characteristics

During the study period, 326 patients with lymphedema underwent LSG, BEI, or CT; of these, the 24 who underwent CT evaluation were analyzed. ISO substage (–5.23 ± 3.67 days), BEI (–3.64 ± 2.67 days), LSG (2.31 ± 4.67 days), and circumference (–5.17 ± 3.75 days) were recorded within 1 week of the CT scanning date. Swelling after surgery was first observed at a median 3 years (range, 3–110 months) after surgery, with CT performed a median 4 years (range, 3–121 months) after initial observation of swelling. None of these patients was classified as ISO substage I or IIIB (Table 1).

**Table 1:** Demographic characteristics.

		Number (%) or mean ± S.E.
Age		57.46 ± 13.41
Sex	Male	0 (0.0)
	Female	24 (100.0)
Side	Right	11 (45.8)
	Left	13 (54.2)
Weight (kilograms)		58.52 ± 10.06
CTNUMBER		109.38 ± 80.12
OPTOCT (months)		82.65 ± 50.74
LETOCT (months)		50.21 ± 42.99
OPTOLE (months)		34.04 ± 32.29
Albumin level in serum (mg/dl)		4.16 ± 0.36
Cancer	Breast cancer	16 (66.6)
	Gynecologic cancer	7 (29.2)
	Other: primary lymphedema	1 (4.2)
History of Radiotherapy	No	6 (25.0)
	Yes	18 (75.0)
ISL sub-stage	IIA	8 (33.3)
	IIB	13 (54.2)
	IIIA	3 (12.5)
History of rehabilitative management	No	7 (29.2)
	Yes	17 (70.8)
History of drug intake	No	7 (29.2)
	Yes	17 (70.8)

CTNUMBER, number of CT scans; OPTOCT, months from operation to CT; LETOCT, months from onset of lymphedema to CT; OPTOLE, months from operation to onset of lymphedema; ISL, International Society of Lymphology

### Correlations between CT reticulation indexes and other factors

**Fig 2:**
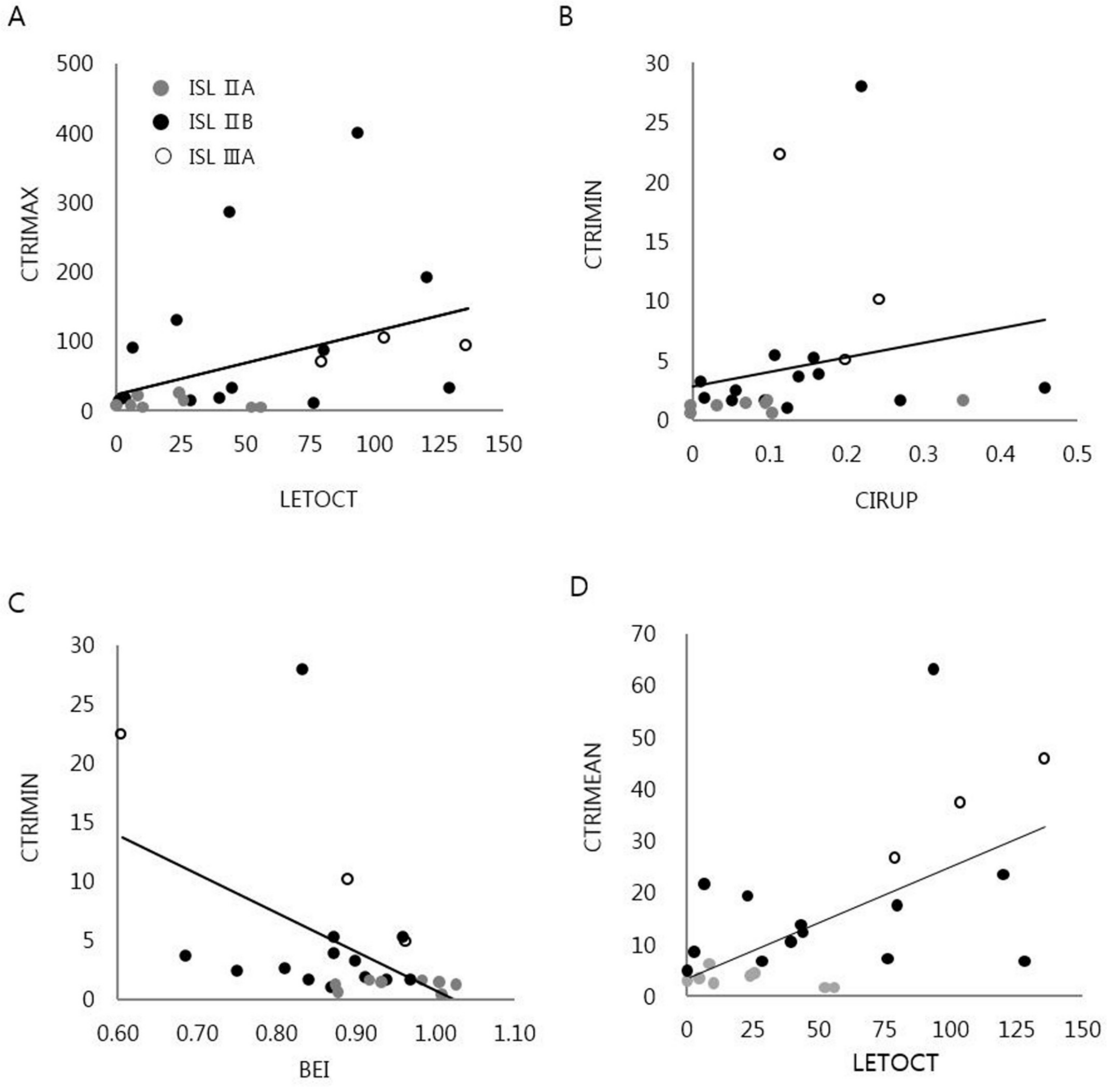
Correlation between the CT reticulation indexes and other clinical measurements. (A) Maximal CT reticulation index (CTRIMAX) showed significant relationship with time from the onset of lymph edema to CT (LETOCT). (B) Minimal CT reticulation index (CTRIMIN) showed significant relationship with difference in the circumference ratio of an affected and nonaffected limb 5 cm proximal to the anatomical landmarks (cm) (CIRUP). (C) CTRIMIN showed significant relationship with 1 kHz-based bio-electrical impedance ratio (BEI) between an affected and nonaffected limb. (D) Mean CT reticulation index (CTRIMEAN) showed significant relationships with LETOCT. Spearman correlation analysis or Mann-Whitney U test.

Mean CTRI (CTRIMEAN) was significantly associated with the time from onset of limb swelling to CT (LETOCT) (*r* = 0.52, *p* < 0.01), but not with the time from surgery to CT (OPTOCT) or to onset of lymphedema (OPTOLE). Similarly, maximal CTRI (CTRIMAX) was significantly associated with LETOCT (*r* = 0.45, *p* < 0.05), but not with OPTOCT or OPTOLE. CTRIMEAN (*r* = 0.86, *p* < 0.01), minimum CTRI (CTMIN) (*r* = 0.79, *p* < 0.01), and CTRIMAX (*r* = 0.68, *p* < 0.01) were all significantly associated with ISL substage. CTRIMIN correlated with 1 kHz-based BEI ratio (*r* = - 0.46, *p* < 0.05) and with proximal SCDR (*r* = 0.45, *p* < 0.05). None of the CTRIs was significantly associated with serum albumin concentration, LSG stage, or distal SCDR (Fig 2).

### Correlations between CT reticulation indexes and epifascial and subfascial lymphatic system dysfunction

**Fig 3:**
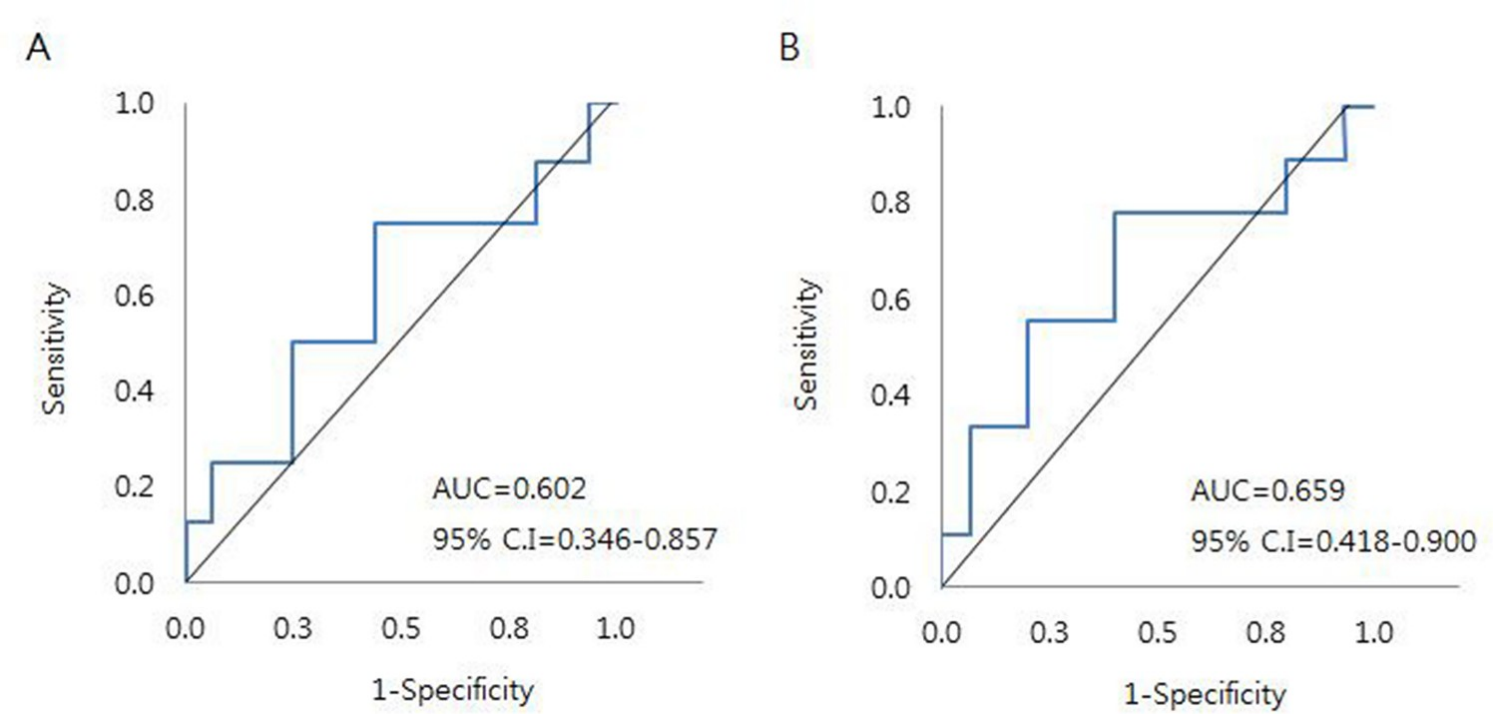
Receiver operating characteristics curve analysis of the effects of CT reticulation indexes on the epifascial and subfascial lymphatic system dysfunction. Maximal value of CT reticulation index can distinguish (A) lymphoscintigraphic stages III and IV from stages I and II and (B) stage IV from stages I, II, and III. AUC. area under the curve; CI, confidence interval.

ROC curve analysis showed that a CTRIMAX cut-off of 17.57 had a sensitivity of 0.78 and a specificity of 0.60 in distinguishing LSG stage IV (no visualization of epifascial or subfascial lymph nodes) and a sensitivity of 0.75 and a specificity of 0.56 in distinguishing LSG stage III (no visible epifascial lymph nodes, but visible subfascial lymph nodes) (Fig 3). However, the areas under the curve (AUC) were 0.66 for the former and 0.60 for the latter.

### Correlations between CT reticulation indexes and other related factors

CTRIs showed no significant correlations with underlying cancers, history of radiotherapy, rehabilitative management, or treatment with anti-edemic drugs (Table 2).

**Table 2:** Comparison of the CT reticulation indexes with other contributing factors.

		CTRIMAX	CTRIMIN	CTRIMEAN
		(Mean ± S.E.)	(Mean ± S.E.)	(Mean ± S.E.)
Breast cancer		82.72 ± 116.98	4.74 ± 8.08	14.29 ± 17.37
Gynecologic cancer		31.11 ± 32.52	3.69 ± 3.11	10.67 ± 8.27
	*p*-value	0.49	0.31	0.77
No Radiotherapy		81.13 ± 116.86	4.02 ± 3.49	12.70 ± 8.57
Radiotherapy		63.09 ± 99.61	4.54 ± 7.64	13.33 ± 16.64
	*p*-value	0.64	0.29	0.49
No rehabilitative management		104.86 ± 146.86	5.89 ± 9.79	18.57 ± 21.17
Rehabilitative management		53.39 ± 72.07	3.84 ± 5.28	12.37 ± 12.89
	*p*-value	0.71	0.85	0.58
No anti-edemic drug		94.59 ± 150.58	5.35 ± 9.93	16.39 ± 21.66
Anti-edemic Drug		57.62 ± 71.95	4.07 ± 5.26	13.27 ± 12.93
	*p*-value	1.00	0.66	1.00

CTRIMAX, maximal value of CT reticulation index; CTRIMIN, minimal CT reticulation index; CTRIMEAN, mean CT reticulation index. Mann-Whitney U test

## Discussion

We hypothesized that digital subtraction from transaxial CT images over the entire limb length might result in three-dimensional estimation of lymphedema-induced fibrosis. To address this hypothesis, we performed an observational, cross-sectional trial in patients with unilateral lymphedema. The key findings of this study were: 1) CTRIs are significantly correlated with lymphedema duration, ISL substage, proximal SCDR, and 1 kHz-based BEI ratio; 2) CTRIMAX shows a sensitivity of 0.78, specificity of 0.60, and AUC of 0.66 in distinguishing subfascial lymphatic system deterioration; and 3) CTRIs showed no significant correlations with history of radiotherapy, rehabilitative management, and anti-edemic drug intake.

Few trials to date have attempted to quantify lymphedema fibrosis. Masson staining of samples from 45 patients with lymphedema showed that stiffness, as determined by the SkinFibroMeter, correlated with histologic fibrosis, but that this correlation was restricted to the skin.[22] Both ultrasonographic indentometry and CT showed quasi-linear correlations with viscoelastic tissue parameters in nine patients. However, these patients had lipodermatosclerotic venous-insufficiency, and fibrosis on CT images was rated using a four-point scale.[23] Moreover, ultrasonography cannot penetrate into deep soft tissues, especially in advanced fibrosis.[24] In contrast, CT can visualize deep tissues. For example, trans-axial CT showed subcutaneous reticulations in a patient with radiation-induced fibrosis.[2] In another study, manual tracing with PiViewSTAR was performed on CT images from two lymphedema patients after extracorporeal shock wave therapy, but that study calculated only the overall area of the epifascial compartment calculated by subtracting the areas of bone and muscle from the total area, instead of calculating fibrosis within epifascial compartment.[25] In other studies, both CT and ultrasonography showed significant correlations between the microscopic degree of fibrosis and histopathologic findings in head and neck cancer patients with dermal lymphatic invasion and in excised adipose tissues from patients with secondary lymphedema, characterized by increases in collagen fibers and fibrous deposits and decreases in elastic fibers.[1, 26] Because immunologic responses can be up-regulated[11] or down-regulated in lymphedema models,[27] leading to dynamic features at the cellular level, attempts to quantify lymphedema fibrosis using macroscopic imaging modalities have failed.

The number of HUs on CT can range from -1000 (air: black) to 1000 (bone: white). The sensitivity of absorptive differentiation can be increased by reducing the width of the window, allowing organs such as the heart and liver to be differentiated.[17] Absorptive values of CT images depend on their relative components. For example, changes in HU can be induced by fatty infiltration, allowing quantitative measurements of sarcopenic muscle degeneration.[28] To date, only one report has described the use of a similar method to assess fibrosis in patients with lymphedema. In that study, epifascial reticulations were quantified by adjusting HU in 16 patients with leg lymphedema. Although trabecular areas were larger in the more-affected than in the less-affected limb, 11 patients (69%) had acute phase lymphedema, and CT venography was performed soon (around 9 days) after swelling first occurred. That study also failed to compare quantification of reticulation with a three-dimensional volume perometry results.[9] Soft tissue HU ranged from 20 to 40.[17] Contrary to the removal of pixels from -50 to 26 HU in the current trial, they removed pixels of -60 to 10 HU. Because the CTRIs in the current study showed high validation on four clinical scales, selection of the upper limit over HU may be critical for clinical validity. The mean number of scans (109.38 ± 80.12) may be important for quantifying the entire length of the limb in three-dimensions, inasmuch as the mean scanning thickness of arms and legs in the current report were 3.8 mm and 5.7 mm, respectively, and 10 mm in an earlier report.[25] Additionally, imaging can be affected by machine parameters or acquisition protocols at individual centers suggesting that, as in the current study, relative ratios should be reported.

In the current trial, all CTRIs significantly correlated with ISL subscale. Ultrasonography in 46 lymphedema patients showed that the echogenicity of fibrosclerosis was increased 64% in patients with moderate edema and 76% in patients with severe edema,[29] and that subcutaneous echogenicity correlated with ISO stage in 35 patients with leg lymphedema. However, 71% of scanning points could not be accurately assessed due to ultrasonographically poor delineation in stage III.[24] However, CTRIs in the current study may not only correlate with ISL substage, but also overcome the limitations of ultrasonography in patients with advanced fibrosis.

CTRIMIN was significantly correlated with 1 kHz-used BEI ratio. Several types of multi-frequency BEI and BEI spectroscopy have been used for interstitial fluid verification in lymphedema.[30, 31] Moreover, lower frequency correlated with more exact measurements.[30] Because a frequency of 0 kHz is not possible, we analyzed BEI at a frequency of 1 kHz, the lowest frequency that can be obtained using commercial devices. However, interstitial hydrostatic pressures vary in lymphedema,[31] including in different compartments (subcutaneous layer, anterior compartment, or posterior compartment) in leg edema,[32] suggesting that maintaining a supine position for 20 minutes may not be sufficient for standardization in the current trial.

Although LSG is performed worldwide, its relationship to clinical staging has not been determined.[21] For example, a study using functional single-photon emission CT in 4328 patients with leg lymphedema reported that LSG did not correlate with ISL stage or with tissue abnormalities,[19] a finding that may explain the lack of correlation between CTRIs and LSG staging in the current trial. Meanwhile, CTRIMAX showed a sensitivity of 0.78 and specificity of 0.60 in assessing subfascial lymphatic system deterioration. However, the AUC is 0.66 and the confidence interval spans 0.5 (0.4 to 0.9); it means at this point the current technique shows very poor separability so that it does not provide any concrete evidence that CTRIMAX can be used as a replacement for LSG staging. Except for lymphangiography, which is rarely used due to extreme pain, only LSG can evaluate the functional state of the epifascial and subfascial lymphatic systems.[21] Although LSG findings were reported to predict future lymphedema, as determined by radioactivity ratio or reduced radio-uptake before and after surgery in the axilla of 201 patients and in 106 patients who underwent breast cancer surgery, those studies did not evaluate both compartments.[33, 34] Because subfascial muscular change did not occur until the epifascial compartment had deteriorated,[9] and because the subfascial lymphatic system is activated if the epifascial system is dysfunctional,[35] early detection of subfascial system deterioration may be essential to avoid progression to an irreversible state. Evaluation of the subfascial lymphatic system in the current study was based on the visualization of popliteal or laterally-located para-cephalic vein lymph nodes.[19, 36] However, visualization may not represent the exact function of these specific lymph nodes. Moreover, a study of a fresh human cadaver showed that previously unnoticed lymphatic interconnections were observed between the epifascial and subfascial systems following lymph node dissection.[20]

In the current trial, CTRIMAX and CTRIMEAN showed significant correlations with LETOCT. Although fibrotic changes depend on the degree of cellulo-molecular responses,[10] lymphedema is highly progressive in nature,[37] such that CTRIs may represent the duration of lymphedema. However, lymphedema does not always occur following cancer surgery, with the time from surgery to initial presentation also varying.[3, 38] Thus, CTRIs did not correlate with OPTOCT and OPTOLE. Furthermore, CTRIMIN in this trial was significantly correlated with proximal, but not with distal, SCDR. Adiposity is greater in proximal than in distal limbs of healthy adolescent girls.[39] Because lymphedema mainly affects adipose tissue,[37] the likelihood of lympho-proliferative inflammation increases as target volume increases, resulting in CTRI being more sensitive in proximal than in distal one limbs. Furthermore, CTRIs in the current study did not correlate significantly with history of radiotherapy, rehabilitative management, or anti-edemic drug intake. In contrast, radiotherapy and the absence of rehabilitative management contributed to enlargement of the epifascial compartment in 511 patients following gynecologic cancer surgery,[40] suggesting that rehabilitative management over time may reduce fibrotic aggravation.[16] Each of these trials, however, evaluated area or volume, not epifascial fibrosis.

The current trial had several limitations. First, it was cross-sectional in design, and included only female patients[41] and those not classified as ISO substage I or IIIB. Instead of showing correlation between the CTRI technique and other clinical measurements, it is important to show its sensitivity to detecting epifascial fibrosis with this approach compared ideally to a gold standard such as a tissue biopsy or if this is not available to some more established method for measuring epifascial fibrosis. Although lymphedema can involve the subfascial compartment,[42] muscular changes[28] were not evaluated. Moreover, the low confidence interval (0.4 to 0.9) and AUC (0.66) means at this point CTRIs cannot be used as a replacement for LSG staging. Despite of claiming the need for early monitoring, the data attempting to address issues related to early monitoring was not presented. Because the number of CT scans per patient varied (109.38 ± 80.12), there may have been variations in standardization of individual patients and increased risks of exposure to radiation. However, the number of scans per patient in our hospital was not larger than the number of scans of other organs, including the chest and abdomen (roughly 170 to 420). Another alternative may be low dose CT using soft tissue characterization.[43, 44] Future trials are required to determine whether a larger number of materials would increase this strength and to correlate these results with the other contributing factors, including obesity,[45] high lymph node retrieval,[40] chemotherapy and age.[3]

## Conclusions

The algorithm for epifascial fibrosis, using digital subtraction from three-dimensional trans-axial conventional CT images, shows promise in providing an additional method to assist in characterizing and monitoring lymphedema patients.
